# Supplementary material for: T cell response to SARS-CoV-2 infection in humans: A systematic review
Source: PLoS One. 2021 Jan 25;16(1):e0245532. doi: 10.1371/journal.pone.0245532 (PMC7833159; doi:10.1371/journal.pone.0245532)
Supplement: S1 File — (DOCX) [file pone.0245532.s002.docx]

**S1 APPENDIX: Sample search strategy**

Database(s): Embase 1996 to 2020 Week 17
Search Strategy:

| **#** | **Searches** | **Results** |
| --- | --- | --- |
| 1 | exp coronavirus/ | 12437 |
| 2 | exp Coronavirus Infections/ | 12279 |
| 3 | ((corona* or corono*) adj1 (virus* or viral* or virinae*)).tw,kw. | 551 |
| 4 | (coronavirus* or coronovirus* or coronavirinae* or CoV or HCoV*).tw,kw. | 15440 |
| 5 | ("2019-nCoV" or 2019nCoV or nCoV2019 or "nCoV-2019" or "COVID-19" or COVID19 or "CORVID-19" or CORVID19 or "WN-CoV" or WNCoV or "HCoV-19" or HCoV19 or "2019 novel*" or Ncov or "n-cov" or "SARS-CoV-2" or "SARSCoV-2" or "SARSCoV2" or "SARS-CoV-2" or SARSCov19 or "SARS-Cov19" or "SARSCov-19" or "SARS-Cov-19" or Ncovor or Ncorona* or Ncorono* or NcovWuhan* or NcovHubei* or NcovChina* or NcovChinese* or SARS2 or "SARS-2" or SARScoronavirus2 or "SARS-coronavirus-2" or "SARScoronavirus 2" or "SARS coronavirus2" or SARScoronovirus2 or "SARS-coronovirus-2" or "SARScoronovirus 2" or "SARS coronovirus2").tw,kw. | 4488 |
| 6 | (((respiratory* adj2 (symptom* or disease* or illness* or condition*)) or "seafood market*" or "food market*" or pneumonia*) adj10 (Wuhan* or Hubei* or China* or Chinese* or Huanan*)).tw,kw. | 1738 |
| 7 | ((outbreak* or wildlife* or pandemic* or epidemic*) adj1 (Wuhan* or Hubei or China* or Chinese* or Huanan*)).tw,kw. | 91 |
| 8 | ("severe acute respiratory syndrome*" or SARS).tw,kw. | 11647 |
| 9 | or/1-8 | 31091 |
| 10 | exp *serology/ | 16162 |
| 11 | exp *antibody/ | 312568 |
| 12 | exp *antibody response/ | 7205 |
| 13 | exp *seroprevalence/ | 5135 |
| 14 | exp *serum/ | 19113 |
| 15 | exp *assay/ | 93624 |
| 16 | exp *immunity/ | 306567 |
| 17 | exp *diagnosis/ | 1200947 |
| 18 | (sero* or antibod* or serum or sera or test or tests or testing or diagnostic or diagnosis or immunity or ''immune response'').tw,kw. | 6175013 |
| 19 | (assay* adj3 sero*).tw,kw. | 6289 |
| 20 | or/10-19 | 7090637 |
| 21 | 9 and 20 | 10106 |
| 22 | limit 21 to english language | 9349 |
| 23 | limit 22 to dc=20200101-20200331 | 451 |
| 24 | limit 22 to dc=20200401-20200428 | 688 |

**S2 FIG.** Data extraction template as used in the review.

**S3 APPENDIX: Approach to critical appraisal of studies included in the review**

There is a wide range of critical appraisal tools (CAT) available to support evidence assessment and synthesis work in public health and policy, but the applicability of these tools in the context of primary work in immunology is limited by (among other limitations):

- An overwhelming focus on interventional studies, rather than those concerned with exposures;
- A relative absence of tools for critical appraisal of laboratory studies, in particular experimental animal models;
- A relative absence of summative tools that enable critical appraisal of varied study types in a standardised way (rather than application of specific checklists for each study design).
- The time and resource-intensity, which limits scope for reproducible, meaningful, and robust appraisal of evidence in the context of a pandemic in which prodigious volumes of evidence are being generated.

These limitations make it very challenging to identify a coherent approach to critical appraisal that allows for evidence quality to be meaningfully compared across multiple study designs. Preliminary scoping work for this review suggested a tendency in the field of immunology to publication of narrative reviews (rather than conventional systematic reviews) without clear description of study selection criteria or critical appraisal methods. For the purposes of this review, we sought to identify a CAT that:

- Was closely related to an existing, validated tool so that it can be presented as a modification of that tool without the need for new and extended validation work;
- Was low burden and quick to use given required speed of turn-around;
- Facilitated high-level comparison of quality across diverse study designs for reporting purposes;
- Would be rapidly interpretable (ideally in the form of a traffic light system or similar) for time-pressed report readers.

Following a review of existing, generic CATs, we selected the Public Health Ontario MetaQAT 1.0 as our base tool ^6^. This is a qualitative critical appraisal tool based around 4 dimensions (relevance, reliability, validity, applicability of research findings). We made a modification to this tool to include additional domains for publication type (pre-print, peer-reviewed etc) and for assay type and validity. Aggregate assessments of evidence strength against the review’s overarching research questions were also made.

**S4 FIG** shows the tool developed from this process. With the exception of the “publication type” domain, all questions required list-based responses in the “assessment” column – i.e. “Yes”, “No” or “Unsure”. The “Additional comments” was used to provide more detailed qualitative appraisal information, principally to address the prompt questions listed in the “Notes/interpretation” column.

**S4 FIGURE.** Adapted MetaQAT template including prompt questions, and weightings applied to each of the main question domains in the tool for the purpose of quality scoring.

**S5 APPENDIX: Full list of included studies**

| ***Paper*** | ***Country*** | ***Publication type*** | ***Study design*** | ***Population*** | ***Relevant Outcomes*** | ***Measurement*** | ***Findings*** | ***Quality assessment*** |
| --- | --- | --- | --- | --- | --- | --- | --- | --- |
| Agrati et al^8^ | *Italy* | Peer reviewed paper | Cohort study | • Human study • Hospital based • 18 SARS-CoV2-infected adults; RT PCR confirmation and patient demographics not stated.  • 9 mild, 9 severe cases included; of these, 4 mild and 4 severe were sampled longitudinally.  • Also, 8 healthy donors used as controls (selection procedure not described) | • T cell activation profile • Myeloid-derived suppressor cell (MDSC) profile | Flow cytometry: Cytoflex (Beckman-Coulter)   MDSC identification with Duraclone Tubes (Beckman-Coulter) | • Compared with mild cases, more severe cases showed lymphopenia, increase in effector T cells, cytotoxic profile of T cells, and expression of CD95 on T cells, as well as higher levels of IL-6. • In severe cases, MDSCs were found to constitute up to 90% of total circulating mononuclear cells while they constituted only up to 25% in mild cases. The frequency of MDSCs was observed to decrease with clinical recovery. | • Sample selection methods not clear • Small sample size • Validity of controls not clear |
| Anft et al^62^ | *Germany* | Pre-print | Cohort study | • Human study • Hospital based • 53 SARS-CoV2 positive adults  • 21 moderate, 18 severe, 14 critical cases | • Quantitative, phenotypic, and functional characteristics of lymphocytes and T-cell subsets by severity group | Flow cytometry: CytoFlex (Beckman Coulter) | • Severe and critical patients (compared with moderate) had lower CD8+ T cells, higher CD4+ T cells, and higher CD4+/CD8+ ratio • An overall loss of activated and differentiated effector T cells was observed in severe and critical cases • The highest responses of spike- specific T cells were in critical cases • Virus-specific CD4+ T cell responses were positively correlated with antibody titres | • Sample selection methods not clear • Small sample size |
| Bai et al^19^ | *China* | Peer reviewed paper | Case series | • Human study • Hospital based • 25 SARS-CoV2 positive children, median age 11 years • 8 asymptomatic, 4 mild, 13 'common' cases | • T cell profile by severity group | Not described | • Most cases had normal CD4+ T cell (23/25) and CD8+ T cell (23/25) counts and all had normal overall lymphocyte counts • There were no statistically significant differences in lab results between severity groups | • Case series design with no controls • Small sample size • Sample selection methods not clear • Assay not stated, therefore validity unknown |
| Bao et al^66^ | *Multiple* | Pre-print | Systematic review with meta-analysis | • Human studies • Hospital based • 5912 SARS-CoV2 positive patients across 35 included reports | • T cell counts by severity group | Various as per included studies; not discussed in detail | • Total numbers of B cells, T cells and NK cells were reduced in all patients with COVID-19 • Severe cases had lower total lymphocytes (1.44-fold), lower CD4+ T cells (2.10-fold), and lower CD8+ T cells (2.00-fold) | • Sampling time points and intervals across included studies were not clear • Assays used by included studies were not described in detail |
| Braun et al^42^ | *Germany* | Pre-print | Case control study | • Human study • Hospital based • 18 SARS-CoV2 positive patients; average age 52.6 years, 72% male  • 7 mild, 5 severe, 6 critical cases; 10/18 ICU-admitted • Also, 68 antibody negative healthy donors, average age 41.9 years, 31% male (selection procedure not described) | • Frequencies and phenotypic characteristics of SARS-CoV-2 S-reactive T cells in COVID-19 patients compared to controls | Flow cytometry: MACSQuant Analyser 16  AIM assay | • Spike-reactive CD4+ T cells were present in most (83%) of COVID-19 patients, as well as in a third of SARS-CoV-2 seronegative healthy donors, though at lower frequencies. • Spike-reactive CD4+ T cells in COVID-19 patients equally targeted N-terminal and C-terminal epitopes of S whereas in healthy donors, spike-reactive CD4+ T cells reacted almost exclusively to the C-terminal epitopes which have higher homology with spike proteins in HCoV • Unlike for controls, spike-reactive CD4+ T cells from cases co-expressed CD38 and HLA-DR, markers of recent in vivo activation | • Small sample size • Baseline differences in case and control groups • Validity of controls not clear |
| Carsetti et al^51^ | *Italy* | Pre-print | Cohort study | • Human study • Hospital based • 36 SARS-CoV2 positive adults • Of positive cases, 20 asymptomatic, 8 mild, 8 severe cases • 28 healthy, SARS-CoV2 negative adult contacts of confirmed cases | • T cell profile in asymptomatic and other severity groups | Flow cytometry: BD FACSLyric (BD Biosciences) | • The circulating pool of T cells was not significantly changed in asymptomatic cases • Increased HLA-DR+ CD4+ T cells were seen in both mild and severe cases, indicating T cell activation • HLA-DR+ CD8+ T cells were increased only in severe cases | • Sample selection methods not clear |
| Chandrashekar et al^30^ | *Animal study* | Peer reviewed paper | Basic Science | • Animal study • Laboratory based • 9 Rhesus macaques initially inoculated and then re-challenged with SARS-CoV2 • 3 further Rhesus macaques as controls, challenged once with SARS-CoV2 | • Cellular immune responses to S proteins, by IFN-γ release • S protein specific CD4+ and CD8+ induction after initial challenge • IFN-γ release at re-challenge | IFN-γ ELISpot (Cellular Technologies Limited) | • Cellular immune responses to spike peptides were demonstrated in most subjects on day 35. There was a trend toward lower responses in lower dose groups.  • Both spike-specific CD8+ and CD4+ T cell responses were observed • Increased IFN-γ responses were observed by day 7 after re-challenge | • Small sample size • Animal model |
| Chen and Sang et al^36^ | *China* | Peer reviewed paper | Cohort study | • Human study • Hospital based • 548 SARS-CoV2 positive patients with known outcome (discharged or deceased) • 345 mild or moderate, 155 severe, 48 critical cases • T cell subset analysis in 141 cases | • T cell profile across severity groups | Flow cytometry: specific device not stated | • Non-survivors showed reductions in peripheral CD3+, CD4+ and CD8+ T cells at the point of admission • For more severe cases there was a trend towards lower CD3+, CD4+ and CD8+ counts, with the percentage of CD8+ T cells significantly lower in severe or critical patients compared to mild or moderate patients • Non-survivors had significantly elevated leukocytes generally, and neutrophils specifically, with an elevated neutrophil/lymphocyte ratio, as well as an elevated platelet/lymphocyte ratio | • Sample selection for T cell assays not clear • Single T cell measurement only (at admission) • Assay not fully described |
| Chen and Zhang et al^35^ | *China* | Peer reviewed paper | Case series | • Human study • Hospital based • 12 SARS-CoV2 positive children, median age 14.5 years  • 20 SARS-CoV2 positive adults as controls | • T cell profile across children and adult cases | Not described | • Paediatric cases had higher total T cells, CD8+ T cells and B cells than adult cases • Most children had normal white cells counts; 2 cases had lymphopenia | • Case series, scope for selection bias • Small sample size • Sampling timings and intervals not clearly described • Cellular assays not described |
| Diao et al^37^ | *China* | Peer reviewed paper | Case control study | • Human study • Multi-centre hospital based • 522 SARS-CoV2 positive patients, both adults and children • 20 were ICU-admitted cases; more in-depth analysis was done on 212 non-ICU cases from one centre of which 151 were mild/moderate, 40 severe, and 13 critical cases (as per Chinese national severity definitions), and also including 8 patient deaths • Also, 40 healthy controls who attended hospital for routine examination during the same period • Detailed T cell phenotypic analysis in 14 patients and 3 controls | • T cell profile across severity groups and age groups • T cell exhaustion phenotypes • Relationship between cytokine concentration and T cell count | Flow cytometry: LSR Fortessa (BD Biosciences) | • Lower T cell counts were observed in COVID-19 cases generally, with relatively lower counts in ICU-admitted cases compared to non-ICU admitted cases, as well as in perished and severe/critical cases compared to mild/moderate cases within the non-ICU group • An age-dependent reduction in T cells was observed, with lowest counts in older groups (of three age categories) • T cells had higher PD-1 and Tim-3 expression in COVID-19 cases, and this increased with clinical progression • TNF-a, IL-6, and IL-10 were significantly increased in COVID-19 cases, with levels in ICU patients significantly higher than in non-ICU patients. Concentration of these three cytokines was negatively correlated with total T cell counts. | • Unclear sample selection of subset undergoing detailed T cell characterisation • Relatively few controls and unclear validity, regarding possible exposure to SARS-CoV2 |
| Dong et al^38^ | *China* | Peer Reviewed paper | Case control study | • Human study • Hospital based • 18 SARS-CoV2 positive adults, mean age 58.39 years, 61.11% male, mean hospital stay 18.22d | • Dynamic T cell profiles for prolonged (≥ 15d) and non-prolonged (<15d) nucleic acid positive patients • Correlation between T cell count and duration of nucleic acid positivity | Flow cytometry: specific device not stated | • Significantly higher T cells and B cells were seen at discharge than at diagnosis for all 15 COVID-19 patients who had both results available • Patients with nucleic acid-positivity for ≥ 15 days had significantly decreased lymphocytes and T cell total and CD4+ and CD8+ subset counts compared to those who remained positive for <15 days • There was a significant negative correlation between lymphocytes and T cells at diagnosis, and the RNA-positivity duration | • Recruited only those with existing flow cytometry data, therefore scope for selection bias • Small sample size • Flow cytometry device not stated |
| Du et al^39^ | *China* | Peer reviewed paper | Cohort study | • Human study • Hospital based • 182 SARS-CoV2 positive children, median age 6 years, 65.9% male  • 43 with history of 'allergic disease' (inc. allergic rhinitis, asthma, atopic dermatitis, urticaria, food/drug allergy);139 with no such history | • T cell profile on admission between those with and without history of allergic disease | Not described | • CD8+ T cell percentage was significantly lower in the non-allergic patients than in the allergic patients  • The lymphocyte subset counts were not significantly different between the allergic and non-allergic patients, or between patients with and without pneumonia • Allergy was not a risk factor for COVID-19 and it did not influence disease severity or clinical course | • Sample selection methods not clear • Small sample size • T cell assays not described |
| Duan et al^43^ | *China* | Pre-print | Case control study | • Human study • Hospital based • 616 SARS-CoV2 PCR or antibody positive adults; median age 64, 57.5% female, median 13 days since symptom onset  • Also, 35 SARS-CoV2 antibody negative but suspected cases (symptoms suggestive of COVID-19); median age 55, 57.1% female, median 8 days since symptom onset | • Lymphocyte profile comparison between the two groups of confirmed COVID-19 cases, and seronegative suspected COVID-19 cases | Not described | • Compared with confirmed cases, the seronegative suspected cases had a significantly higher lymphocyte count and relative proportion of CD8+ lymphocytes | • Sample selection methods and, SARS-CoV2 PCR testing process, and therefore designation of groups not clear • Days from symptom onset substantially different between two groups, significant potential for bias • T cell assay not described • Overall methodology not clear and high potential for bias |
| Gallais et al^52^ | *France* | Pre-print | Cohort study | • Human study • Hospital based • Cluster of 7 families with 9 COVID-19 recovered index cases, median age 45, 50% male, median symptom duration 7 days  • Also, 8 antibody negative contacts of index cases (6/8 were symptomatic within 1-7d of index cases) and 10 antibody negative healthy donors (not known to be exposed to COVID-19 cases) were used as controls.  • Blood samples collected 47-69 days post-symptom onset | • Virus-specific T cell responses between index cases, contacts and controls | IFN-γ ELISpot | • All index patients showed SARS-CoV2-specific IFN-γ responses to at least 4 epitopes (S1, S2, N and M).  • 6/8 contacts showed virus-specific responses to at least one epitope (structural and/or accessory); 5/6 of these had been symptomatic • The frequency of reactive T cells in 5 contacts was similar to index patients and higher than in healthy controls • Half of the controls showed some T cell responses to 1-2 SARS-CoV2 epitopes (mostly S2), but of much lower magnitude than cases and contacts • All participants, except one control, showed T cell responses against HCoV (229E and OC43) | • Unclear PCR testing processes, may have affected differentiation of index and contact cases • Small sample size • Validity of healthy donor controls not clear |
| Ganji et al^40^ | *Iran* | Peer reviewed paper | Case control study | • Human study • Hospital based • 25 SARS-CoV2 positive patients admitted to ICU • 25 healthy controls of similar age and sex distribution (selection procedure not described) | • T cell profile in COVID-19 cases compared to healthy control • T cell subset protein expression by mean fluorescence intensity | Flow cytometry: BD FACSCalibur (BD Biosciences)  Mean fluorescence intensity (MFI) evaluation | • The difference in CD4/CD8 ratio, CD4+ T cell frequency, CD8+ T cell frequency, and CD4+ MFI was not significant between cases and controls • There was a significant increase of CD8+ MFI in cases vs controls • WCC, lymphocyte, and platelets were significantly reduced in ITU cases vs controls | • Sample selection methods not clear • Small sample size • Only ICU-admitted patients, no lower severity cases included • Validity of healthy controls not clear |
| Gimenez et al^9^ | *Spain* | Peer reviewed paper | Case series | • Human study • Hospital based • 19 SARS-CoV2 positive cases, 6 suspected cases; medial age 62 years, 56% male, T cell sampling at a median of 27 days from symptom onset | • Virus-specific T cell profiles across two groups • Association between virus-specific T cells and antibodies | Flow cytometry: FACScanto (BD Biosciences) Intracellular cytokine staining assay | • Ten patients (40%) had responses targeting S1 and M proteins  • SARS-CoV-2-reactive IFN-γ-CD8+T cells developed at comparable rates irrespective of disease severity. However, the two patients who died did not have detectable responses. • No correlation was found between levels of reactive CD8+ T cells and titres of spike-specific antibodies amongst confirmed cases | • Sample selection methods not clear • Small sample size • No control group • Assay validity unclear (adapted protocol from CMV assay) |
| Grifoni et al^10^ | *USA* | Peer Reviewed paper | Case control study | • Human study • Hospital based • 20 SARS-CoV2 positive adults, median age 44, 55% female • 70% mild, 20% moderate, 10% severe cases • 20 unexposed controls, median age 31, 65% female (samples collected prior to SARS-CoV2 emergence) • Samples collected a median of 26 days post symptom onset | • Virus-specific T cell responses between cases and controls • Association between T cells and antibody titres • Correlation between CD4+ and CD8+ T cells | Flow cytometry: specific device not stated Activation induced marker (AIM) assay Intracellular cytokine staining assay | • Higher spike-specific CD4+ and CD8+ T cell responses were detected by AIM in cases vs controls • Spike-specific CD4+ T cell responses positively correlated with the magnitude of anti-RBD IgG levels  • Anti-spike IgA levels also correlated with spike-specific CD4+ T cells  • Virus-specific CD4+ and CD8+ T cell responses correlated well with each other | • Small sample size • Baseline differences between cases and controls |
| Hartman et al^41^ | *Animal study* | Pre-print | Basic Science | • Human study • Hospital based • 6 adult male African Green monkeys; average age 3.5 years • Inoculated with SARS-CoV2 via aerosol (4/6) or mucosal (2/6) exposure | • Dynamic cellular response profiles in SARS-CoV2 challenged animals by route of infection | Flow cytometry: specific device not stated | • Relative proportions of proliferating CD4+ and CD8+ T cells increased transiently between 2-11 days after infection in most subjects, with sustained increases in proliferating CD8+ T cells in the mucosally-infected group. | • Animal model, validity of inoculation routes not clear • Small sample size • No control group |
| He et al^11^ | *China* | Peer reviewed paper | Case control study | • Human study • Hospital based • 204 SARS-CoV2 positive patients  • 135 non-severe, 69 severe cases (as per Chinese national COVID-19 pneumonia classification criteria) • Subset of 15 severe (7 recovered and 8 died) and 14 non-severe patients included in a longitudinal analysis | • T cell profile in severe vs non-severe cases • Dynamic changes in T cells over course of admission (subset of 15 severe and 14 non-severe patients) • Sensitivity and specificity of T cell subset counts for disease severity | Not described | • Significant increases in WBC cells and neutrophils, and decreases in lymphocytes were observed in severe cases; also CD3+, CD4+, CD8+, were significantly lower in severe cases  • From the onset of pneumonia, T cells gradually increased during admission in non-severe patients, and remained higher than in the severe group. In the improved subgroup of severe patients, T lymphocytes began to increase after 15 days and normalised after 25 days. In contrast, T lymphocytes in the dead sub-group of severe patients continued to fall until death. • Counts of CD3+, CD4+, and CD8+ T cells showed high sensitivity and specificity for disease severity | • T cell assay not described • Small sample size for longitudinal measurements |
| Hu et al^53^ | *China* | Pre-print | Cohort study | • Human study • Hospital based • 211 SARS-CoV2 positive patients; average age 47.5, 39.2% | • Association of immune cell counts and cellular ratios, with presence of antibodies | Not described | • There was no significant difference in WCC, T and B lymphocytes, CD4+ and CD8+ T cells, and CD4+/CD8+ ratio between the IgM / IgG seropositive and seronegative groups. | • Sample selection methods not clear • T cell assay not described |
| Jiang et al^13^ | *China* | Peer reviewed paper | Cohort study | • Human study • Hospital based • 32 confirmed cases (13 mild, 10 severe, 9 critical) and 18 age- and sex-matched controls. | T cell profile over the disease course (and by severity) | Flow cytometry: FACSCanto II flow cytometer (BD, USA). | • CD8+ T cells in COVID-19 cases, particularly critical cases were highly activated with possibly higher cytotoxic potential  • The absolute numbers of lymphocytes, total T cells, CD8+ T cells, and NK cells were restored in the remission group • Expression of CD38, HLA-DR on CD8+ T cells decreased in the remission group indicating reduced CD8+ T cell activation and cytotoxic potential | • Selection process for study participants and controls unclear • Study received research ethics committee approval but process for individual participant consent are unclear |
| Jiang et al^12^ | *China* | Peer reviewed paper | Case control study | • Human study • Hospital based • 103 SARS-CoV2 positive patients; median age 46, 56.3% males • 86 mild/moderate, 17 severe ICU-admitted cases • Also, 13 healthy individuals with no infectious disease diagnosis and no known exposure to COVID-19 were used as controls (selection procedure not described) | • T cell profile in cases vs controls and across severity groups • T cell counts over disease course and by nucleic acid positivity (subset of 23 cases only) | Flow cytometry: Cytomics FC 500 (Beckman Coulter) | • Cases had a slight decrease in CD4+T cells and significant decrease in CD8+T cells, with raised CD4/CD8 ratio • Severe cases showed significant decreases in CD3+T, CD4+T, and CD8+ T cells counts compared to mild/moderate patients • There was no significant difference in proportion of Treg cells between mild/moderate and severe cases • Counts of CD3+, CD4+ and CD8+ T cells dramatically recovered in most patients who became RNA-negative but there was no significant difference for persistently RNA-positive cases | • Sample selection methods not clear • Small sample size for longitudinal measurements • Validity of controls not clear |
| Juno et al^44^ | *Australia* | Pre-Print | Case control study | • Human study • Community based • n=41 patients who had recovered from COVID-19, median age 59 (IQR:54-65), of which 43% were female, and 27 healthy adult controls • Participants sampled a median of 36 days post symptom onset | B and T cell response according to disease severity Plasma neutralisation activity | Activation Induced Marker Assay + ELISA | • Frequency of T follicular helper (TFH) cells specific to HKU1 (an endemic human coronavirus) was higher amongst COVID-19 convalescents than uninfected controls • Expanded populations of spike-specific memory B cells and circulating (c)TFH cells detected • High plasma neutralisation activity was also found to be associated with increased spike-specific antibody, but notably also with the relative distribution of spike-specific cTFH subsets. | • Sample size small • Inclusion and exclusion criteria are unclear |
| Kang et al^14^ | *South Korea* | Peer Reviewed paper | Case control study | • Human study • Hospital based • 12 cases, of which 4 severe and 8 mild cases; 5 healthy control volunteers | Leucocyte response according to disease severity | Flow cytometry | • No significant difference in CD4 and CD8 cell counts between groups in first week. In second week, CD4+ count 396.5 ± 101.9 in the severe group vs. 684.3 ± 82.0 in the mild group (p=0.062). CD8+ T cell, 250.9 ± 50.1 in the severe group vs. 445.0 ± 76.1 in the mild group (p=0.174)  • Higher indicators of T cell proliferation observed in severe group vs mild group over the course of the study. • Activated T cells tended to be higher in the severe group than in the mild group at the third week, especially in terms of PD-1 expression (mean ± SEM, the severe group compared with the mild group. • Frequencies (%) of perforin and granzyme B expression in both CD4+ and CD8+ T cells were higher in the severe group than in the mild group. | • Very small sample size • Process for selection of participants unclear • Controls were included in the study, but it is unclear whether they were matched to the main participant group |
| Laing et al^54^ | *UK* | Pre-Print | Cohort study | • Human study • Hospital based • 63 hospitalised COVID-19 cases, 55 healthy controls. Cased were sampled variously on the day of admission or between 3-6 days post-admission. | B and T cell response according to disease severity | Flow cytometry | • T cell cytopenia observed across all disease categories, with decreases in CD4 and particularly CD8 T cells.  • Gamma delta T cells severely depleted across all disease categories. CD4 TH17.1 substantially depleted in severe patients • Putatively terminally differentiated CD8 TEMRA cells were 'significantly' depleted across patients • Consistent with their activation, there were approximately 10-fold increases in the percentage of CD4 and CD8 T cells in either G1 or S-G2/M phases of the cycle in patients, in contrast to healthy controls where over 98% of cells comprise G0 cells in transit. | • Relatively small sample size |
| Le Bert et al^55^ | *Singapore* | Pre-Print | Cohort study | • Human study • Hospital based • PBMC samples from 24 individuals who had recovered from mild-severe COVID-19, 23 individuals who had recovered from SARS-CoV-1 and 18 samples from people not exposed to SARS CoV-2 | T cell response against nucleocapsid proteins and NSP proteins on SARS-CoV-1/2 | IFN-γ ELISpot assay cross-referenced with intracellular cytokine staining (ICS) | • A clear population of NP specific CD4 and/or CD8 T cells were detectable in 7/9 subjects tested. • COVID-19 convalescents developed T cells specific to regions that were also targeted by T cells of SARS recovered subjects • NP-specific T cells were detected in some of the SARS-CoV-1/2 unexposed individuals, although the pattern of T cell reactivity was different. NSP7/13-specific T cells were detected in 9 out of 18 (50%) SARS-CoV-1/2 unexposed donors. | • Small sample size • Method of participant and control selection unclear (assumed to be convenience-based) - no evidence that controls were matched |
| Li et al^15^ | *China* | Peer reviewed paper | Cohort study | • Human study • Hospital based • 127 paediatric cases enrolled between January and March 2020 with confirmed COVID-19 (confirmed using RT-PCR) • Median age 6 years (IQR: 1-9 years, range: 2 months to 15 years), ratio of M:F = 1.3:1. 2 of the 127 patients ended up in ITU. 125 of the patients were discharged during the lifetime of the study. | Lymphocyte subsets | Flow cytometry: FACSCanto) | • Low CD4+/CD8+ ratio observed in 18.3% of participants • CD4+CD25+ T lymphocyte <5.0 % was statistically associated with clinical diagnosis of pneumonia (OR 1.93, 95% CI 1.04-3.61, P=0.038) | • Method of participant selection unclear (assumed to be convenience-based) • Assays stated but not clear whether these were validated • No detail on mode by which participant consent was sought (in a paediatric study) |
| Liu and Fang et al^63^ | *China* | Pre-print | Cohort study | • Human study • Hospital based • 340 confirmed COVID-19 cases (310 discharged, 30 deaths), median age 57 | T cell subset counts | Flow cytometry: FACSCalibur flow cytometer (BD Biosciences) | • T lymphocyte subsets – total T cells, T helper cells and suppressor T cells – were significantly lower among patients who ultimately died than those who survived to discharge. • Multivariate logistic regression modelling (outcome = death) identified predictors including age (OR 1·05, p 0·04), underlying disease status (OR 3·42, p 0·02), Helper T cells on the log scale (OR 0·22, p 0·00), and TH/TS on the log scale (OR 4·80, p 0·00). | • No control group recruited • Unclear how participants were selected (assumption is that this was convenience-based) • T cell testing approach status but no detail given on validation status or use of controls |
| Liu and Han et al^45^ | *China* | Pre-print | Case control study | • Human study • Hospital based • 128 patients in total - 37 non-severe SARS ('persistently positive') CoV-2 patients + 37 non-severe SARS CoV (positive on assessment) patients + 54 healthy controls  • Mean age of participants (SD) = 5.09 (4.71); male n = 23, female n =17 • Median age 53, 67% of the sample were male | CD3+, CD4+ and B cell counts | Flow cytometry: FACS Aria III cytometer (BD bioscience, USA) | • Among persistently positive (PP) patients, absolute numbers of CD3, CD4 and NK were significantly higher than in the positive on assessment (PA) group.  • Compared to those persistently positive with a negative PCR after a further 7 days, those with a positive result had a significantly lowerCD3, CD4, CD8 and B cell count (p=0.001, =0.005, =0.003 and =0.003 respectively).  • Furthermore, amongst those who converted from persistently positive to negative, their CD3, CD4, CD8 and B cell counts increased significantly between the last positive and first negative tests (p=0.001, =0.002, =0.009 and =0.008 respectively).  • No significant differences between the PP group and the healthy control group, but patients from both groups had increased numbers of CD3+T cells, CD4+T cells, and NK cells compared to those from the PA group. In addition, PA patients had significantly lower frequency of B cells compared with healthy subjects. | • Sampling based on clinical presentation – but controls were included and were matched (age and sex)  • Assay validity unclear |
| Liu and Li et al^16^ | *China* | Peer reviewed paper | Cohort study | • Human study • Hospital based • 27 "mild" and 13 "severe" COVID-19 cases with a mean age of 48.7, and 62.5% female overall (narrow majority male in the severe category) | T cell subset counts and percentages | Lymphocyte test kit (Beckman Coulter Inc., FL, USA).   Human Th1/2 cytokine kit II (BD Ltd., Franklin lakes, NJ, USA). | • There were significant decreases in the counts of T cells, especially CD8+ T cells, as well as increases in IL-6, IL-10, IL-2 and IFN-g levels in the peripheral blood in the severe cases compared to those in the mild cases.  • T cell counts and cytokine levels in severe COVID-19 patients who survived the disease gradually recovered at later time points to levels that were comparable to those of the mild cases. | • Small sample size  • No control group recruited • Unclear how participants were selected (assumption is that this was convenience-based) |
| Liu and Liao et al^17^ | *China* | Peer reviewed paper | Case control study | • Human study • Hospital based • 76 patients, 46 with mild disease, 30 with severe | CD4+, CD8+ count Interleukin levels | Not described | • CD4 and CD8 count significantly lower in severe disease than in mild disease (P<0.001 for both).  • IL-2R, IL-8 and IL-6 significantly higher in severe disease than in mild disease (p = 0.022, 0.026 and 0.012 respectively).  • Lymphocyte, CD4+T lymphocyte, and CD8+T lymphocyte counts in the severe group were significantly lower than they were in the mild group (Z= -5.889,-4.932, and-5.505, respectively; p<0.001). |  |
| Liu and Long et al^20^ | *China* | Peer reviewed paper | Cohort study | • Human • Hospital based  • 39 hospital patients with lymphocyte subsets measured on admission. Median age 53yrs (IQR: 41-61), 51% female, 46.2% severe or critical infection. 38.5% had comorbidities. | T cell subset counts | Not described | • T cells, CD4+ T cells, and CD8+ T cells were all statistically higher in patients who had a mild infection, timely (<5 days PSO) hospitalization, and fast recovery (all p<0.05). • Patients whose RT-PCR turned negative within 14 days after onset showed higher levels of both T and B cells (p<0.05). • CD8 cells were higher in those without comorbidities than in those with (p=0.046) | • No control group recruited • Unclear how participants were selected (assumption is that this was convenience-based) • T cell testing approach status but no detail given on validation status or use of controls |
| Liu and Wang et al^18^ | *China* | Peer reviewed paper | Case series | • Human • Hospital based  • 154 cases among adults • Stratified by severity: moderate group (total 49, male 26 and female 23), severe group (total 61, male 34 and female 27), critical group (total 44, male 24, female 20) • The average age of the moderate group, the severe group, and the critical group is 63 ± 13, 63 ± 14, and 65 ± 15 y, respectively. | T cell counts | Flow Cytometer BNII (Becton Dickinson). | • CD3+ and CD4+ lower than normal range in 78% and 76% of participants respectively.  • CD3+, CD4+ and CD8+ values lower in critical group compared to severe group (statistically significant - p<0.001), but no difference was found between the moderate and severe groups | • No control group recruited • Unclear how participants were selected (assumption is that this was convenience-based) • T cell testing approach status but no detail given on validation status or use of controls |
| Luo et al^21^ | *China* | Peer reviewed paper | Case control study | • Human • Hospital based • 1018 diagnosed COVID-19 patients from a retrospective clinical cohort presenting to two centres in China - divided into a survivor cohort (n=817) and a non-survivor quasi control (n=201) • Mean age 61, 51% male overall, but 66% in the non-survivor group; some 36% of the study sample overall had diagnosed hypertension | T cell subsets; cytokine levels | CLIA: Immulite 1000, DiaSorin Liaison, Italy,or Cobas e602, Roche Diagnostics.  Flow cytometry. | • CD3 +, CD4+ and CD8+ counts significantly lower in non-survivors than in survivors. The absolute count of CD8+ was below 100 cells/μL in non, less than half of the total number of surviving cells. • CD8+ cell count lower 96.89 vs. 203.98 cells/μL,P<0.001 (for difference) for non-survived vs survived • CD8+ T cell counts <165 cells/μL independently associated with mortality (OR=5.930; 95%CI, 3.677-9.562;P<0.001), after adjusting for confounding factors including age, sex, and underlying diseases | • Risk of bias in selection of participants based on presentation to hospital setting • No matching performed for control group • T cell measurement approach described but nature of controls used unclear |
| Mann et al^56^ | *UK* | Pre-print | Cohort study | • Human • Hospital based • Recruitment of participants from 4 acute centres in Manchester. 73 patients in total, of which 49 could be stratified based on disease severity • Control group of 18 females and 9 males recruited a neighbouring university and NHS trusts (age range 28-69) • Overall median age was 61 (IQR 51-71)and 63% were male | T cell counts; subsets; cytokine levels | Flow cytometry - in-house. | • Reduced T cells in acute phase (across CD4 and CD8 with slight decrease in CD4+ T cells in severe cases • Both T cell subsets showed signs of activation in cases, more striking in CD8+ T cells, did not track with disease severity, and highly variable • Higher perforin expression in CD8+ T cells in cases compared to healthy individuals  • Increase in T cells prior to discharge (linked with recovery) in 70% of cases irrespective of severity • Other data only presented in charts • Unclear if statistical analysis performed on cases vs controls | • Risk of bias in selection of participants based on presentation to hospital setting • No matching performed for control group • T cell measurement approach described but nature of controls used unclear |
| Marcos-Jimenez et al^57^ | *Spain* | Pre-print | Cohort study | • Human study • Hospital based • 276 patients stratified according to illness severity in accordance with WHO scheme • 19 healthy controls | T cell subsets and counts | Flow cytometry - in-house. | • CD8+ decreased in severe cases, but CD4 similar in cases and controls • cTFH proportion significantly increased with severity: median 0.51 in healthy donors to 1.7 % in severe (this difference was statistically significant) | • Participant inclusion and exclusion criteria are not clearly stated • Control group was recruited but no information on characteristics of this group or mode of recruitment is provided • T cell measurement approach described but nature of controls used unclear |
| Mazzoni et al^22^ | *Italy* | Peer reviewed paper | Cohort study | • Human study • Hospital based • 30 adult patients with SARS-CoV2 confirmed by RT-PCR; 30 adult controls (healthy) also recruited • Median age of the patients was 70 years (range 36-85 years), the mean age was 65.9 years. 60% of the cases were male. The mean age was 67.9 years for men and 63 years for women. • Immunological analysis performed on average 9.2 days PO | T cell subsets and counts | Flow cytometry - in-house. | • Absolute CD3+ lower in cases than controls (including lower subsets of CD4+, CD8+ and CD56+) (statistically significant) • Reduction more pronounced in CD8+ (cytotoxic) than CD4+ (helper) • CD4+/CD8+ ratio higher in cases than controls (statistically significant) • Absolute numbers of CD3+ lower in ICU compared to non-ICU, only for CD4+ subset (not CD8+ or CD56+) | • Risk of bias in selection of participants based on hospital presentation |
| Minervina et al^64^ | *Russia* | Pre-print | Basic Science | • Human study • Hospital based  • 2 adult patients, 1 male and 1 female, recruited who were RT-PCR positive for SARS-CoV2 and were symptomatic | CD4+ and CD8+ count; measurements of cell reactivity | Flow cytometry: FACS Aria III | • Identified groups of T cell clones which contracted from day 15 to 45 with recovery and further group which expanded from day 15 to day 37 • Both donors developed IgG response by day 30 and remained positive to end of follow up (day 45) | • Very small sample size • Method for selection of participants unclear • No controls included • Method for measurement of T cell response unclear |
| Neidleman et al^73^ | *USA* | Pre-print | Case series | • Human study • Hospital based  • 4 adult patients who tested positive for SARS-CoV2 via RT-PCR and recovered from mild infection, 2 uninfected blood donors as controls | CD+ and CD8+ counts T cell phenotyping | Flow cytometry - in-house. | • Spike specific CD4 and CD8 cells producing IFN present in cases and not controls, and were phenotypically diverse • Different characteristics of CD8 cells identified and reported in detail in paper - narrative | • Very small sample size • Method for selection of participants unclear • Method for measurement of T cell response unclear |
| Ni and Tian et al^24^ | *China* | Peer reviewed paper | Case series | • Human study • Hospital based • 27 adult patients, 14 male and 13 female, age 33-83 and median 60 years, all of whom were confirmed to have COVID-19 by RT-PCR • All defined as having severe pneumonia, with requirement for supplemental O2 | IL‐1β, IL‐2R, IL‐8, IL‐10, and TNF‐α levels T cell counts and subsets | • IMMULITE 1000 Immunoassay system (Siemens Healthcare Diagnostics Products Limited) Electrochemiluminescence immunoassay (Cobas E601; Roche, Basel, Switzerland)  Flow cytometry | • Pre-treatment T lymphocytes were decreased in 76.2% of participants (mean 720+/- 328), CD4+ decreased in 57.1% (mean 472+/-280) and CD8+ decreased in 85.77% (mean 219 +/-113) - all statistically significant • Post treatment T lymphocytes in normal range for all participants - (mean 1080+/-341), CD4 mean 678+/-255, CD8 mean 355+/-138) - all statistically significant | • Risk of bias inherent in case series based on clinical presentation alone (and given absence of a control group) • No formal statistical evaluation of immune response according to potential confounders was made |
| Ni and Ye et al^23^ | *China* | Peer reviewed paper | Case control study | • Human study • Hospital based • 14 adults with confirmed COVID-19 (age range 32-68) and 6 healthy controls (samples from the US) - all adults who had had the disease were convalescents | T cell counts and subsets | Flow cytometry - in-house. | • No difference in percentages of T cells between cases and controls • The numbers of NP-specific T cells (indicating that the development of neutralizing antibodies may be correlated with the activation of anti-viral T cells) (statistically significant) | • Small sample size • Method for selection of participants unclear although controls were recruited and are adequately described |
| Odak et al^58^ | *Germany* | Pre-print | Cohort study | • Human study • Hospital based  • 30 adult patients hospitalised (24 male, 6 female) with COVID-19 • 60 age and gender-matched healthy controls • Age range 39-91 | • T cell counts - total and subsets | BD TruCount™  Flow cytometry: BD FACS Lyric + FCSExpress (De Novo Software). | • Compared to healthy controls, mild-disease and severe-disease COVID19 cases displayed generalised lymphopenia and reduced T-reg cell numbers.   • Lower counts of NK, NKT, and CD8+ T cells were observed in cases with severe but mild disease  • Decrease in the proportion of memory/effector CD4 T cells in severe disease compared to mild disease   • Decrease in naïve and increase in effector/memory and central memory CD4 and CD8 T cells observed in convalescent cases | • Sample selection and inclusion criteria not clearly described • No control for confounders beyond age • No report of an Fc block and live-dead marker in counts panel  • Appears that the comparison between memory and naïve T cells was performed on non-Tregs only |
| Ouyang et al^25^ | *China* | Peer reviewed paper | Case control study | • Human study • Hospital based  • 11 patients in total, of which 5 mild, 6 severe/critical • Median age 63, 5 male, 6 female, 3 with an underlying chronic condition | • T cell counts • Plasma cytokine levels | Luminex bead based MILLIPLEX® assay for the cytokines T Cell proportions - CyTOF based analysis | • IL-10 level significantly varied with disease progression and treatment.   • T cell subsets were generally reduced in severe patients, CD8+ T cell counts much slower to normalise than CD4+ | • REC approved but unclear about consent • Selection process unclear, analysis unclear in parts  • No control group • Small sample size  • Lack of clarity in relation to statistical results, including number of tests performed |
| Patterson et al^65^ | *USA* | Pre-print | Non-randomised controlled trial | • Human study • Hospital based  • 10 critically ill individuals • All had significant pre-existing co-morbidities | • T cell percentages • CD4/CD8 T cell ratio • Plasma cytokine levels | Customized 13-plex bead-based flow cytometric assay (LegendPlex, Biolegend, Inc) CytoFlex flow cytometer (Beckman Coulter Life Sciences, Indianapolis, IN). Kaluza version 2.1 software. | • Levels of IL-6 significantly higher in critically ill patients compared to patients with mild or moderate COVID-19 • Post Leronlimab treatment, marked restoration of CD8+ T cells and normalization of CD4/CD8+ T cell ratio observed | • Sample selection and inclusion criteria not clearly described • Small sample size • Characteristics of controls not provided |
| Payen et al^59^ | *France* | Pre-print | Cohort study | • Human study • Hospital based  • 15 adult COVID-19 inpatients followed longitudinally (for overall changes over time) (specific n=5 for function T cells), ICU patients • Median age 66 [IQR 60-72], 80% male | • T cell subsets over time  • Functionality of T cells over time | Flow cytometry: BD FACSLyric™ Clinical System (BD Biosciences)  Navios flow cytometer for IFN (Beckman Coulter) | • CD8+ counts fell to a minimum by days 11-14 post-onset of symptoms (p=0.03) with recovery thereafter • Later nadir for CD4+ (days 19-23, not statistically significant) observed, with no significant change in the CD4/CD8 ratio overall throughout the follow-up period • N specific CD4 T cells less numerous than S specific CD4 T cells • A decrease in CD4 and CD8 T cells was observed in severe patients, however no disruption of the CD4/CD8 ratio is shown (polyfunctional SARS-Cov-2-specific CD4 T-cells were present and functional, whereas virus-specific CD8 T-cells were less frequent and not efficient). Isolation of SARS-COv2-specific T cells showed that after in vitro stimulation CD4 T cells were more capable of secreting cytokines (IFNg, TNFa, IL-2) with higher TNFa, IL-2 positive cells. | • Sample selection and inclusion criteria not clearly described • Small sample size • No controls |
| Peng et al^46^ | *UK* | Pre-print | Case control study | • Human study • Hospital based  • 42 adult patients hospitalised with COVID-19 who subsequently recovered, including 28 mild and 14 severe cases - days PO 42.5 for mild and 41.5 for severe Mild - median age 53.8, IQR 47.6-60.9, 60% male, n=28 Severe - median age 60.6, IQR 44.9-74.1, 64% male, n=14 • 19 controls - median age 46, 8 male | • Frequency of T cells, correlated with disease severity • T cell epitope characteristics | BD LSR Fortessa (BD Biosciences) flow cytometer and FlowJoTM v.10 software  IFN-γ ELISpot assays | • Positive correlations (all statistically significant) between spike specific, RBD and NP specific antibody titres and overall T cell and spike specific T cells • A higher magnitude and broader breadth of overall T cell responses observed in severe cases compared to mild, in particular responses to spike, membrane, ORF3 and ORF8proteins (all statistically significant) • More T cell responses detected to spike- and M/NP found to be mediated by CD8+ (rather than CD4+) T cells in mild compared to severe  • Functional memory responses identified and cytotoxic potential CD8 cells identified • Six immunodominant epitope clusters identified and showed low similarity to epitopes from other coronaviruses. | • Selection of controls not clearly described |
| Qin et al^26^ | *China* | Peer reviewed paper | Case control study | • Human study • Hospital based  • 452 hospitalised patients with COVID-19, 286/452 clinically severe infection.  • Median age 58yrs (IQR 47-67), 52% men, 44% had chronic disease.  • Lymphocyte subsets were analysed in 44 patients with COVID-19 on admission | • Lymphocyte subset response according to severity. | Phorbol 12-Myristate 13-Acetate (PMA)/ionomycin-stimulated lymphocyte function assay   Flow cytometry: FACSCanto flow cytometer | • T cells counts were lower in severe cases (461.6 vs 663.8/μL; P = .027) when compared with the non-severe group. This pattern occurred for multiple T cell subtypes. • The total number of B cells, T cells, and natural killer (NK) cells were significantly decreased in pa-patients with COVID-19 (852.9/μL), which was more evident in the severe cases. • Both helper T (Th) cells (CD3+,CD4+) and suppressor T cells (CD3+,CD8+) in patients with COVID-19 were below normal levels, and the decline in Th cells was more pronounced in severe cases. | • No controls used • Small sample size for lymphocyte assessments |
| Shomuradova et al^47^ | *Russia* | Pre-print | Case control study | • Human study • Mixed hospital and community   • 31 COVID-19 Convalescent patients, - samples collected days 14-49 • Control group of 14 health donors -samples collected pre-COVID | • T cell response in convalescent patients | IFNγ secretion assay (Milteny biotech)  IFNγ ELISpot assay (ELISpot kit) ELISA (theromoscientific) | • Some healthy COVID-19-naive donors had T cells specific to SARS-CoV-2 antigens and in particular spike-protein • No clear association observed between T-cell response magnitude and HLA genotype of the donor, time post disease onset, disease severity or age.  • A mild correlation between the magnitude of T-cell and humoral response was observed (for anti-RBD IgG and CD8+ T-cell response r=0.386 p=0.0321)  • The magnitude of CD8+ and CD4+ response magnitudes were interdependent • Among convalescent donors, significantly more CD4+ cells in expressed HLA-DR and CD38 - a similar tendency for CD8+ T cells was observed, although the difference was not significant.  • Most SARS-CoV-2 specific CD4+ belonged to the TCM subpopulation, whereas most CD8+ T cells displayed a TTE and TEM phenotype | • Sample selection process not clearly described • Small sample size |
| Thieme et al^60^ | *Germany* | Pre-print | Cohort study | • Human study • Hospital based  • 28 COVID-19 patients with moderate (8), severe (10) and critical (10) manifestations • All critical patients were male; age range for the sample as a whole 26-91 | • T cell counts and percentages | CytoFlex flow cytometer (Beckman Coulter) | • Stimulation with M-protein overlapping peptide pools induced the highest frequencies of reactive CD4+ T cells. • In comparison to S- and N-reactive CD4+ T cells , higher frequencies of M-reactive CD4+ T cells were detected. These cells expressed effector molecules e.g. interleukin (IL)-2, interferon γ (IFNγ), tumour necrosis factor α (TNFα), and granzyme B (GrzB) • Exposure to the N-protein induced the lowest responses | • Sample selection and inclusion criteria not clearly explained • Small sample size • No controls used • Unclear presentation of methodology and findings |
| Vabret et al^3^ | *Multiple* | Peer reviewed paper | Narrative review | • Methodology for selecting papers for review not disclosed | • T-cell response | N/A | • Drastically reduced numbers of both CD4 and CD8 T cells observed in moderate and severe COVID-19 cases.   • Severity of lymphopenia, most prominent for CD8 T cells in patients requiring intensive care, appears to correlate with COVID-19-associated disease severity and mortality   • Now widely documented that a decrease in peripheral blood T cells is associated with disease severity and inflammation .  • Multiple studies find raised numbers of activated CD4 and CD8 T cells, trending toward an exhausted phenotype in persistent COVID-19 infection, accompanied by ongoing and upregulated expression of inhibitory markers and potential reduced polyfunctionality and cytotoxicity. | • Search strategy not described • Critical appraisal not discussed • Quantitative synthesis not attempted |
| Varnaitė et al^48^ | *Sweden* | Pre-print | Case control study | • Human study • Hospital based  • 20 hospitalised COVID-19 patients, 7 with co-morbidities.  • Blood samples collected a median of 5 days POS • 7 healthy control donors | • T-cell response | BD Trucount Tubes (BD Biosciences) BD Accuri Plus flow cytometer BD LSRFortessa flow cytometer  BD FACSDiva Software (BD Biosciences) | • Decreased absolute numbers of lymphocytes (CD45+) and T cells (total CD3+ and CD3+ CD8+ cells) observed in cases compared to controls • No significant decrease of B cells (CD19+) or CD4+ T cell observed.  • Higher frequencies of activated CD4 and CD8 T cells measured | • Sample selection process not clearly described • Small sample size • Assay validity not clear |
| Wang and Su et al^28^ | *China* | Peer reviewed paper | Case control study | • Human study • 12 patients, split by clinical condition at presentation i.e. mild, n=4; severe, n=5; and critical, n=3 • Mean age was 58 years old, 42% were men, and 57.1% were women; 42.9% had chronic diseases such as hypertension, diabetes, and cardiovascular disease | T cell counts | Mass cytometry (CyTOF) | • Proportions of B cells, CD4+CD8+double-positive T cells, naïve CD4+T cells, and TGF-β+CD28-naïve CD4+T cells where generally increased in infected patients versus health donors • CD8+T cells, irrespective of their the effector, naïve, or memory phenotype, declined over the duration of disease progression. The same pattern was observed for NK cells, monocytes, myeloid-derived suppressor cells (MDSCs), and regulatory T cells (Tregs) - that is increase the observed during progression from mild to severe infection followed by decline when progressing to a critical condition.  • Proportions of dendritic cells (DCs), macrophages, CD4+T cells, and TGF-β+CD28-naïve CD8+T cells were found to be higher in the mild compared to the severe group | • Sample selection process for both cases and controls not clearly described • Small sample size • Validity of controls not clear • No statistical analysis done |
| Wang, Hou and Luo et al^27^ | *China* | Peer reviewed paper | Case control study | • Human study • Hospital based  • 65 SARS-CoV2 positive patients, mean age 57, 57% male • Illness classified as mild (n=30), severe (n=20), and extremely severe (n=15) | • T cell counts | Flow cyometry: FACSCanto flow cytometer (BD Biosciences). FACSCanto clinical software (BD Biosciences). | • Absolute numbers of CD4+ T cells, CD8+ T cells and cells declined with increased illness severity of illness • Markers of activation, e.g. HLA-DR and CD45RO, expressed on CD4+ and CD8+ T cells were raised in severe and extremely severe compared with mild cases | • Small sample size for more severe disease groups • No controls used • Application of inclusion criteria not fully described |
| Wang, Hou and Yao et al^29^ | *China* | Peer reviewed paper | Case control study | • Human study • Hospital based  • 157 COVID-19 patients  • Controls - 95 survived and 62 deceased patients.  • No difference in groups between age and gender | • T cell counts • CD4/CD8 T cell ratio | Flow cytometry - no further details provided | • Non-survivors had lower CD4+ counts only evident in middle and late stages of disease compared to survivors. • A lower CD4/CD8 ratio was also observed non-survivors. | • Sample selection process not clearly described • Methodology not fully described • Statistical analysis not done |
| Weiskopf et al^49^ | *Netherlands* | Pre-print | Case control study | • Human study • Hospital based • 10 SARS-CoV2 positive patients; average age 58.9 years, 60% male  • 10/10 with previous ARDS and ICU admission, later 2 deceased and 1 discharged • Also, 10 unexposed controls (samples collected prior to SARS-CoV2 emergence) | • Virus-specific T cell responses, phenotypes and cytokine production in severely ill COVID-19 patients compared with unexposed controls | Flow cytometry: BD FACSLyric (BD Biosciences) AIM assay | • CD4:CD8 ratios were increased in COVID-19 patients recovering from ARDS when compared to controls • SARS-CoV-2-specific CD4+ and CD8+ T cells were detected in 100% and 80% of COVID-19 patients, with activation defined by cell surface expression of CD69 and CD137.  • Strongest T cell responses were directed to the surface glycoprotein (spike, S) • SARS-CoV-2-specific CD4+ and CD8+ T-cells appear in blood of ARDS patients in the first two weeks post onset of symptoms, and their frequency increases over time • SARS-CoV-2-specific CD4+ T-cells in blood typically had a central memory phenotype (based on CD45RA and CCR7 expression), whereas the majority of virus-specific CD8+ T-cells was identified as CCR7- effector memory (TEM) or terminally differentiated effector (TEMRA) • Low levels of SARS-CoV-2-reactive T-cells were detected in 20% of the unexposed controls | • Sample selection methods not clear • Small sample size |
| Wen et al^31^ | *China* | Peer reviewed paper | Case control study | • Human study • Hospital based  • 10 COVID-19 patients (divided into early recovery stage (ERS) group and late recovery stage (LRS) group) • All males • Age range 30–80 years old, with a median of 58 years old in ERS, a median of 49 years old in LRS and a median of 55 years old in heathy controls | • T cell counts • CD4/CD8 T cell ratio | No details provided on methods of T cell enumeration | • CD4+ T cells and CD8+ T cells decreased significantly and expressed high levels of inflammatory genes in the ERS group | • Sample selection process not clearly described • Small sample size • No controls used |
| Xu et al^32^ | *China* | Peer reviewed paper | Case control study | • Human study • Hospital based  • 187 adult patients hospitalised with COVID-19 | • T-cell response | Flow cytometry - no further details provided | • All patients, at admission, had significantly decreased lymphocyte counts (median = 0.88, IQR 0.55-1.27 mg/L) • All patients, particularly the severely and critically ill including dead patients, displayed a significant reduction in T cell subsets counts with increased concentrations of SAA and CRP compared to normal value • The median counts of lymphocyte, CD3+ T-cell, CD4+ T-cell, and CD8+ T-cell in critically-ill patients, were almost decreased to a third of median counts detected in mildly-ill patients, and equal to nearly half of the median counts observed in severely-ill patients • 62 patients had T cell subsets measured more than once, including mild-ill (n=18), severe-ill (n=22) and critically-ill (n=22). After 5-14 days post admission, T cell subsets counts slightly increased than above that observed at disease onset.  • Total T cell counts <500/µl, CD3+ counts <200/µl, CD4+ or CD8+ counts <100/µ as well as B cell counts <50/µL, were significantly associated with risk of in-hospital death, however this is only on univariate analysis | • Sample selection process not clearly described |
| Yang et al^50^ | *China* | Pre-print | Case control study | • Human study • Hospital based  • 38 COVID-19 cases. Median age 39.06±4.26 years , 23 men, and 15 women • 18 healthy controls | • T cell counts • Plasma cytokine levels | Flow cytometry: FACSCanto II flow cytometer (BDBiosciences) | • Based on CD25 expression, mild activation of CD4+ T cells was detected in COVID-19 patients with a stronger activation in CD8+ T cells observed.  • No correlation with age was measured | • Sample selection process not clearly described • No characterisation of study population • Methodology not fully described |
| Zhang and Zhou et al^61^ | *China* | Pre-print | Cohort study | • Human study • Hospital based  • 222 Patients. Median age 62. 48.2% male and 39.2% patients with severe disease. 194 recovered from diseased and 59 recovered by anti-viral supported therapy.  • Serum samples collected at admission and convalescence (35 days) | • T-cell counts • Cytokine levels | Human Cytokine Standard Assays panel (ET Healthcare, Inc., Shanghai, China) and Bio-Plex 200 system (Bio-Rad, Hercules, CA, USA) | • Severe patients had higher proinflammatory cytokines levels including IL-2, IL-6 and IL-10 where detected in patients with severe disease compared to those with non-severe disease. • Severe patients with severe disease and high neutrophil-to-lymphocyte ratio displayed higher proinflammatory cytokines levels including IL-2, IL-6 and IL-10, and decreased CD4+ T cell count (p<0.05). | • Selection of cases not clearly described • Sampling process and intervals not consistent across the study • Assay validation unclear |
| Zhang et al^33^ | *China* | Peer reviewed paper | Case series | • Human study • Hospital based • 6 children varying in age from 7-131 months • 5 healthy controls | • T cell percentages | Aria II flow cytometer (BD), Flow Jo v10 software (BD) | • Percentage of CD3+, CD4+, and CD8+ T cells were comparable between cases and controls.  • No difference in CD45RA and CCR7 expression in CD4+ and CD8+T cells was observed between four infected paediatric cases and five controls | • Sample selection process not clearly described • Small sample size • Methodology not fully described • No controls used |
| Zhou et al^34^ | *China* | Peer reviewed paper | Cohort study | • Human study • Hospital based  • 17 patients. Disease progression measured at day 7 post admission. 11 women and 6 men between 18 and 70 years. • 5 pts with 'aggravated disease' and 12 with 'non aggravated'  [aggravation: temperature is higher than previous measurement, respiratory symptoms more severe, and  lesion(s) on lung CT is larger in size than previously measured] | • T-cell counts | No details provided on methods of T cell enumeration | • Correlation was found between total lymphocyte and CD4 T-cell count • In the "aggravation" group of patients, CD4+T cell count was significantly lower compared to the "non-aggravation" group (P=0.034). No significant difference was observed between the 2 groups in relation to the CD8+T cell count. • CD4+T cell count was positively correlated with total lymphocyte count (r=0.940, P<0.0001) | • Small sample size • Use of non-standard clinical classifications • Assays not clearly described |

**END OF SUPPLEMENTARY MATERIAL**
